# Impact of packed red blood cells versus fresh whole human blood on the growth of nutritionally variant streptococci and *Streptococcus anginosus* group during verification of the BACT/ALERT VIRTUO blood culture system

**DOI:** 10.1128/jcm.01987-24

**Published:** 2025-03-31

**Authors:** Heather Glassman, Jasmine Ahmed-Bentley, Rebecca Buckman, Mark Carbungco, Colleen Fletcher, Kim Sy, Joyce Wong, Charlene Porter, Claudine Desruisseaux, Valery Lavergne, Suefay Liu, Anthony Lieu, Marthe K. Charles

**Affiliations:** 1 Division of Medical Microbiology & Infection Control, Vancouver Coastal Health Authority 25469, Vancouver, British Columbia, Canada; 2 Department of Pathology and Laboratory Medicine, University of British Columbia 8166, Vancouver, British Columbia, Canada; Children's Hospital Los Angeles, Los Angeles, California, USA

**Keywords:** blood culture, verification and validation, automated system, spiked human blood sample

## LETTER

The most common continuous-monitoring blood culture systems in clinical microbiology laboratories include BACT/ALERT (bioMérieux, France), BACTEC (BD, USA), and VersaTREK (ThermoFisher, USA). In 2024, Vancouver General Hospital’s microbiology laboratory began verification of the BACT/ALERT VIRTUO prior to a transition from the BACTEC FX. During the verification, there was a failure of the VIRTUO to detect nutritionally variant streptococci and some strains of the *Streptococcus anginosus* group in the BACT/ALERT bottles.

The verification protocol was a parallel contrived blood culture study using expired packed red blood cell (PRBC) units from the local Transfusion Medicine department. PRBCs were chosen for low cost, availability, guideline concordance, and laboratory experience (1). The study included 20 frozen, archived clinical bacterial and yeast isolates. The respective aerobic, anaerobic, and pediatric bottles for each system (BACT/ALERT FA Plus, FN Plus, PF Plus and BACTEC Plus Aerobic/F, Lytic Anaerobic/F, Peds Plus/F) were inoculated with 3 mL (pediatric bottles) or 5 mL (aerobic/anaerobic bottles) of PRBC. Approximately 30 cfu/bottle (10–100 cfu/bottle) bacterial or yeast suspension was added and confirmed with colony count plates. The bottles were incubated simultaneously at 35°C until positive or a maximum of 5 days in each of six VIRTUO instruments. Positive bottles were sub-cultured, and identification was confirmed by MALDI-TOF MS (Bruker, USA). Growth of the expected organism and time-to-detection (TTD) were compared between VIRTUO and BACTEC.

We expected 100% concordance of growth between VIRTUO and BACTEC. However, the *Granulicatella adiacens* and *Streptococcus constellatus-1* isolates did not grow in the VIRTUO or grew with greatly extended TTD compared to BACTEC, particularly in the aerobic and pediatric bottles (Table 1). This discrepancy persisted in four different PRBC units despite repeat testing and higher organism concentrations. Terminal subcultures of the original aerobic and pediatric bottles were negative for all *Granulicatella adiacens* replicates but positive for 5/6 aerobic and 6/6 pediatric bottles for *Streptococcus constellatus*. Additional clinical isolates were added, and the same pattern was observed with *Abiotrophia defectiva*, whereas *Streptococcus constellatus*-2 or *Streptococcus anginosus* grew as predicted. Subsequently, the original isolates, *Streptococcus constellatus*-1 and *Granulicatella adiacens*, were tested with fresh whole human blood obtained from consenting laboratory volunteers, which then grew with comparable TTD to BACTEC.

**TABLE 1:** Laboratory verification results for BACT/ALERT VIRTUO compared to BACTEC^*c*^

Organism	Bottle type	Blood type	cfu/bottle	Average VIRTUO TTD^*a*^ (h:min)	Number of VIRTUO instruments^*b*^ (*n*)	BACTEC TTD (h:min)	Difference in TTD between VIRTUO and BACTEC (h:min)
*Granulicatella adiacens*	Aerobic	PRBC	18	NG	6	17:53	NG VIRTUO
			50	NG	6	15:39	NG VIRTUO
			>1,000	55:47	3	11:38	+44:09
				NG	3		NG VIRTUO
		Whole blood	61	18:34	6	17:53	+00:41
		None	39	NG	6	ND	ND
		None + FOS	13	NG	6	ND	ND
	Anaerobic	PRBC	18	20:38	6	16:34	+04:04
			50	18:15	6	14:39	+03:36
			>1,000	14:30	6	11:18	+03:12
		Whole blood	42	13:32	6	16:34	−03:02
	Pediatric	PRBC	18	NG	6	15:04	NG VIRTUO
			50	NG	6	13:57	NG VIRTUO
			>1,000	101:00	3	10:17	+91:13
				NG	3		NG VIRTUO
		Whole blood	59	18:34	6	15:04	+03:30
		None	23	NG	1	ND	ND
		None + FOS	13	NG	1	ND	ND
*Streptococcus constellatus*-1	Aerobic	PRBC	52	114:00	2	15:45	+98:15
				NG	4		NG VIRTUO
			57	102:00	6	45:49	+56:11
			>1,000	68:30	6	22:09	+46:21
		Whole blood	31	21:24	6	ND	ND
		None	23	NG	1	ND	ND
		None + FOS	13	86:00	1	ND	ND
	Anaerobic	PRBC	52	44:25	6	19:03	+25:22
			57	39:31	6	17:09	+22:22
			>1,000	29:26	6	13:09	+16:17
		Whole blood	ND	ND	ND	ND	ND
	Pediatric	PRBC	52	110:24	4	15:13	+95:11
				NG	2		NG VIRTUO
			57	101:00	1	48:49	+52:11
				NG	5		NG VIRTUO
			>1,000	73:18	6	23:39	+49:39
		Whole blood	47	21:26	6	15:13	+06:13
		None	23	NG	1	ND	ND
		None + FOS	13	NG	1	ND	ND
*Streptococcus constellatus*-2	Aerobic	PRBC	66	20:35	6	19:21	+01:14
	Anaerobic		78	25:28	6	14:51	+10:37
	Pediatric		57	23:26	6	16:12	+07:14
*Streptococcus anginosus*	Aerobic	PRBC	33	13:28	6	13:31	−0:03
	Anaerobic		17	39:39	6	14:12	+25:27
	Pediatric		45	13:25	6	12:59	+00:26
*Abiotrophia defectiva*	Aerobic	PRBC	51	NG	6	20:40	NG VIRTUO
	Anaerobic		42	29:19	6	18:10	+5:19
	Pediatric		38	NG	6	19:00	NG VIRTUO

^*a*^ Average TTD is calculated as the mean TTD of the VIRTUO instruments that detected growth. In total, six VIRTUO instruments were introduced into the laboratory.

^*b*^ The number of VIRTUO instruments that detected growth and from which the average was calculated. For example, “55:47 *n* = 3” denotes the average was taken from three VIRTUO instruments, and the other three did not detect growth within 5 days.

^*c*^ NG, no growth at 5 days incubation; ND, not done. FOS, BD BACTEC FOS culture supplement.

To investigate the reason for the discrepancies, additional testing of *Streptococcus constellatus*-1 and *Granulicatella adiacens* was performed without blood and with or without BD BACTEC FOS (BD, USA) supplement (containing hemin, bovine serum albumin, and nicotinamide adenine dinucleotide). In all cases, they failed to grow or grew with extended TTD.

The literature review reveals a lack of published seeded blood culture studies, including nutritionally variant streptococci or the *Streptococcus anginosus* group and PRBC with the VIRTUO. Interestingly, Yarbrough et al. (2) noticed failures to detect *Kingella kingae* and extended TTD for *Bacteroides fragilis* with VIRTUO and PRBC.

PRBCs differ from whole blood in several ways, including leukoreduction, platelet and plasma removal, and different additive solutions (3). General PRBC manufacturing differences have been shown to affect bacterial survival and growth rates (4). This study is limited by the small number of isolates. Results could vary between strains and may reflect a difference in growth factors in the VIRTUO and BACTEC broths, an inhibitory effect of the PRBC, or other factors.

These data suggest that whole blood should be preferred for simulated blood culture verification studies with the VIRTUO. Laboratories that choose to use PRBC should be aware that this may lead to a delay or failure to detect some strains of the *Streptococcus anginosus* group and nutritionally variant streptococci.
